# An Evaluation of Healthcare Use and Child Morbidity 4 Years After User Fee Removal in Rural Burkina Faso

**DOI:** 10.1007/s10995-018-02694-0

**Published:** 2018-12-22

**Authors:** David Zombré, Manuela De Allegri, Robert W. Platt, Valéry Ridde, Kate Zinszer

**Affiliations:** 1 Department of Social and Preventive Medicine, University of Montreal, Montréal, Canada; 2 University of Montreal Public Health Research Institute – IRSPUM, Pavillon 7101 Avenue du Parc C.P 6128 Succursale C, local, 3224, Montréal, QC H3C 3J7 Canada; 3 Institute of Global Health, Medical Faculty, Heidelberg University, Heidelberg, Germany; 4 Departments of Pediatrics and of Epidemiology, Biostatistics, and Occupational Health, McGill University, Montréal, Canada; 5 IRD (French Institute For Research on sustainable Development), CEPED (IRD-Université Paris Descartes), Universités Paris Sorbonne Cités, ERL INSERM SAGESUD, Paris, France

**Keywords:** User fee removal, Child health, Access to healthcare, Inequalities, Burkina Faso

## Abstract

*Objectives* Increasing financial access to healthcare is proposed to being essential for improving child health outcomes, but the available evidence on the relationship between increased access and health remains scarce. Four years after its launch, we evaluated the contextual effect of user fee removal intervention on the probability of an illness occurring and the likelihood of using health services among children under 5. We also explored the potential effect on the inequality in healthcare access. *Methods* We used a comparative cross-sectional design based upon household survey data collected years after the intervention onset in one intervention and one comparison district. Propensity scores weighting was used to achieve balance on covariates between the two districts, which was followed by logistic multilevel modelling to estimate average marginal effects (AME). *Results* We estimated that there was not a significant difference in the reduced probability of an illness occurring in the intervention district compared to the non-intervention district [AME 4.4; 95% CI  1.0–9.8)]. However, the probability of using health services was 17.2% (95% CI 15.0–26.6) higher among children living in the intervention district relative to the comparison district, which rose to 20.7% (95% CI 9.9–31.5) for severe illness episodes. We detected no significant differences in the probability of health services use according to socio-economic status [χ^2^ (5) = 12.90, p = 0.61]. *Conclusions for Practice* In our study, we found that user fee removal led to a significant increase in the use of health services in the longer term, but it is not adequate by itself to reduce the risk of illness occurrence and socioeconomic inequities in the use of health services.

## Significance

*What is already known on this subject?* Increasing financial access to health services by providing free care is recommended to improve access which will improve health outcomes. However, whether such improvements can be maintained in the longer term is an issue that has received less attention and the effect on the likelihood of morbidity is not clear.

*What this study adds?* The results suggest that, 4 years after its launch in Dori, Burkina Faso, a user fee removal intervention was not associated with a reduced probability of the occurence of the most common child illnesses, but is still effective in maintaining the use of child health services. User fee removal contributes to reduced geographic inequity in access to health centres although is equity neutral in terms of access to health services according to socioeconomic status.

## Introduction

Despite significant progress in recent years, child health remains a major concern in Burkina Faso (Batana et al. 2012). Low rates of use of health services and high levels of morbidity among children continue to characterize many communities, especially the poor (Batana et al. 2012; INSD 2012). More than 1 in 9 children die before the age of 5 with 70% of these deaths being largely preventable (Batana et al. 2012). While seeking prompt and appropriate care is critical to help reduce child morbidity (Rutherford et al. 2010) in Burkina Faso, only 1 in 2 children access formal healthcare (INSD 2012). Several factors that influence healthcare seeking behaviour include gender, health-related cultural beliefs, traditional beliefs about causes of illness, trust in traditional medicine, distance, and the difficulty of accessing care given the terrain (Ahorlu et al. 2006; Batana et al. 2012; Colvin et al. 2013; INSD 2012). Critically, user fees remain the main barrier to seeking care, due to the lack of health insurance resulting in a high proportion of households not being able to pay the cost of the care when confronted with illness (Batana et al. 2012; INSD 2012).

There is the widespread belief that scaling up health-care financing reforms such as free care policies have the potential to increase coverage for several important child health interventions (Bassani et al. 2013), contributing to the reduction of infant morbidity (Rutherford et al. 2010). This is the context in which over the last few years several countries have implemented free care policies for children under 5 years of age (Ridde and Morestin 2011).

Several mechanisms may explain the relationship between user fee removal, the use of healthcare, and child health outcomes. Conceptually, user fee removal act as an enabling factor, allowing users to make choices about their use of public healthcare services (Robert et al. 2017). By removing financial barriers to healthcare access, individuals and households will change their health seeking behavior as services become more affordable (Robert et al. 2017; Sen 2002). Individuals will use healthcare services more often and more regularly and will seek care promptly (Giedion et al. 2013), which will maintain the use of health services (Giedion et al. 2013; Yates 2009). Health is then improved through the regular use of health services (Giedion et al. 2013; Mosley and Chen 1984; Robert et al. 2017; Rutherford et al. 2010). However, despite the achievement of healthcare access via reduction of financial barriers, the improvement in health will depend on quality of health services that need to be improve and maintained (World Health Organization, Organisation for Economic Co-operation and Development, & Bank 2018). Other individual, community, and environmental-level factors including socioeconomic status (SES) and illness severity may also modify the use of free health services (Mosley and Chen 1984; Ridde et al. 2013a, b; von Lengerke et al. 2014).

Prior studies have generally shown a positive association between user fee removal and the use of health services in low-income countries (Lagarde and Palmer 2011; Ridde and Morestin 2011). In Burkina Faso, Sierra Leone, Uganda, and Mali, user fee removal have been associated with significant increases in the use of health services among children under 5 (Druetz et al. 2015; Johri et al. 2014; Ridde et al. Ridde et al. 2013a, b; Zombré et al. 2017b). User fee removal was also associated with a 4.4% drop in the frequency of self-reported illnesses among children under 5 in Uganda (Deininger and Mpuga 2004), and a 40% decrease in the prevalence of children with febrile illness in Mali (Johnson et al. 2013). In Burkina Faso, user fee removal has been associated with an increased use of health services across all socioeconomic groups, irrespective of health needs and distance to the health center (Ridde et al. 2013a, b) while the equity in access according to wealth only improved in the medium and long-term in Jamaica (Li et al. 2017). Conversely, in a randomized community trial in Ghana, no significant association was found between user fee removal and the prevalence of severe and moderate anemia (Ansah et al. 2009; Powell-Jackson et al. 2014). However, it should be noted that most evaluations were conducted early, within 1 year of implementation, and almost all studies focused on health seeking behaviours rather than on health itself as the main outcome (Ansah et al. 2009; Bassani et al. 2013; Druetz et al. 2015; Ridde and Haddad 2009; Ridde et al. Ridde et al. 2013a, b). The few studies addressing the impact of user fee removal on socioeconomic inequalities in service utilization have shown mixed results (Li et al. 2017; Ridde et al. 2013a, b), and the possibility of maintaining a strong association with the use of services and reduction in morbidity and inequalities beyond one year have not yet been assessed.

In this paper, we extend previous studies to examine whether living in a setting where user fees were removed, is associated with a reduced probability of the occurrence of an illness and with an increased likelihood of health service use among children under 5 years in the Sahel region of Burkina Faso. We also examine whether distance to health services, SES, and illness severity moderate these relationships. Drawing from previous studies (Bassani et al. 2013; Ridde et al. 2013a, b; Ridde and Morestin 2011) and conceptual frameworks (Giedion et al. 2013; Mosley and Chen 1984; Robert et al. 2017), we hypothesize that years following user fee removal implementation, children residing in areas with free care will experience fewer illnesses and demonstrate a higher use of health services as compared to children residing in areas without free care. We also hypothesize that the implementation of user fee removal contribute to reducing inequalities in health services utilization according to SES, the distance to the health center, and health needs.

## Materials and Methods

### Context and Intervention

This study was conducted in 2012 in 2 of the rural districts in the northern region of Sahel, Burkina Faso. This is one of the country’s most disadvantaged regions with over half of the population living on < 1 USD per day. In the Sahel region, the rate of severe anaemia in children (20%) is the highest, vaccination coverage (65% for all vaccinations) and health-centre utilisation are the lowest with only 32% of children with fever using healthcare (INSD 2012). In order to improve access to healthcare for children under 5, the districts of Dori and Sebba in the Sahel region, implemented a pilot user fee removal intervention for children under 5, with support from the German NGO Help. Before the intervention, patients visiting a health centre paid for a consultation ($0.20), for drugs (varying costs depending on the prescription), and for care under observation ($0.60/day) if they were hospitalised in the health center (Ridde et al. 2013a, b). The pilot user fee removal program covered the costs of medications, in addition to services and hospitalization in the district of Dori and Sebba. The districts of Gorom and Djibo maintained standard user fees for child health services. In addition to user fee removal, the intervention also included activities related to social mobilisation, health education, improvement of service quality (training, supervision) and financial monitoring (Ridde et al. 2013a, b). The user fee removal integrated within the health system and rolled-out across the country due to a national policy which began in April 2016.

### Study Design

The study was cross-sectional in design, based upon data collected years after the intervention onset in one intervention district (Dori) and in one comparison district (Gorom). The intervention was rapidly deployed in September 2008 in a humanitarian aid context funded by the Humanitarian Aid Service (ECHO) of the European Commission, therefore it was not feasible to conduct a baseline assessment in the comparison district. On the basis of this quasi-experiment, we chose Dori as intervention district and Gorom as comparison district given their proximity and similarities. Two longitudinal studies on the effects user fee removal on the use of child health services (Zombré et al. 2017a) and assisted childbirth (Nguyen et al. 2018) showed almost similar patterns of service utilization in both groups before intervention.

We used data collected by means of a cross-sectional household survey conducted between July and August 2012 in the intervention and comparison villages. Sampling in the two districts relied on a stratified two-stage random probability sample following the WHO’s Expanded Program on Immunization (EPI) Cluster Survey Design (Ridde et al. 2013a, b). During the first stage, we randomly selected villages in the two groups for inclusion in the study with probability proportional to their population size, to ensure that each household of the survey population had the same chance of being included in the sample in order to increase the efficiency. During the second stage, we enumerated households in each selected village and randomly selected 30 to 40 households (Milligan et al. 2004). We included all children under 5 years of age (6–59 months) in each household and we interviewed the mother or primary caregiver on the occurrence of illness episodes, occurring a severe illness episode, and related health service utilization in the 30 days prior to the survey date. In addition, we interviewed the head of the household to obtain information on household socio-economic, demographic, and environmental characteristics.

### Outcome Variables

In the literature on the assessment of the effect of health interventions on health outcomes, including studies evaluating the impact of user fee removal, researchers generally rely on morbidity measures such as: self-reported illness episodes during the previous weeks (Ansah et al. 2009; Hatt and Waters 2006; Ridde et al. 2013a, b), self-reported number of sick days (Nguyen and Wang 2013), or infectious disease-related outcomes (Ansah et al. 2009; Quimbo et al. 2011). Our measures of morbidity relied on the standard approach used in DHS morbidity questionnaire using a 30-day recall period for the most common symptoms in child, related to malaria, diarrhea and diarrhea, pneumonia (WHO 2013). We defined three self-reported binary outcomes measured for each child: (1) an illness episode; (2) a severe illness episode; and (3) use of health services for the reported illness episode. An illness episode was considered severe if the child had stopped or reduced his usual activities and/or presented signs of danger, such as one or several of following symptoms: unable to eat or drink, had repeated vomiting, was pale, lethargic or unconscious, had convulsions, had difficulties breathing, had rapid breathing.

### Exposure

As the intervention was implemented at the district level, meaning that the healthcare facility fees were removed for all children within the same village from the same district, we defined exposure status based on geographical location. If a child resided within the district where the user fee removal had been ongoing for years, the child was considered exposed, versus living in the district with regular user fees (non-exposed).

### Covariates

In each of our three regression models, we included: age, gender, mother’s education (if attended school or not), household size, sleeping under bednet, household SES, village-level SES, distance to health services (0 = 0 < 5 km; 1 = 5–10 km; 2 = 10 km and plus), and illness type and severity. We used an asset index as a proxy of household SES, which was computed using principal component analysis (Filmer and Scott 2007). Household ownership of specific assets were combined with characteristics of the dwelling (Filmer and Scott 2007). Households were categorized as belonging to the lowest SES category (≤ 1st quintile), middle SES category (≥ 2nd < 5th quintile), and highest SES (≥ 5th quintile). We obtained village level wealth by aggregating household wealth scores to the village level to differentiate between low, middle, and high SES status. We captured households’ and health facilities’ GPS coordinates and calculated household straightline distance to the nearest health facility and the mean distance to access to health service at village level. We categorized distance into 3 groups: (< 5 km, 5–10 km, > 10 km).

### Statistical Analysis

#### Estimating the Propensity Scores

As the intervention was not randomly assigned, characteristics of villages, households, and children living within the intervention district were likely to be different to those from the comparison district. To ensure that the joint distributions of covariates sufficiently overlapped and were balanced across intervention and comparison groups, we relied on generalized boosting modeling (GBM) propensity scores methods (McCaffrey et al. 2004). The GBM is a non-parametric technique that estimates the propensity score for the binary treatment indicator using a flexible estimation method that can adjust for a large number of pre-treatment covariates using complex functional forms (Lee and Little 2017; McCaffrey et al. 2004). In practice, for each child in the intervention district, the propensity score was equivalent to the conditional probability of being similar to a child in the comparison district, as a function of individual, household, and village level confounders (Rosenbaum and Rubin 1983). We fit the generalized boosting model (GBM) using the package *gbm* for R software version 3.5.1 (Lee and Little 2017; McCaffrey et al. 2004). All covariates which were potentially related to the exposure and outcomes variables were included in the boosting models (Lee and Little 2017). We ran the GBM algorithm using the standardized mean difference stopping rule to identify the iteration that minimizes the average standardized mean differences or standardized effect size (std.eff.sz) defined as the intervention group mean minus the control group mean divided by the pooled sample standard deviation (McCaffrey et al. 2004).

#### Estimating Main Effects

We first tested a two-level random intercept logistic regression model, taking into account the clustered data structure (children within households and households within villages). We used stabilization procedures (Robins et al. 2000) to correct the influence from children with extreme weights by multiplying the inverse probability of treatment weights (IPTW) by a constant, equal to the expected value of receiving the treatment relative to what the child actually received (Robins et al. 2000). We then incorporated the estimated propensity score in our regression models as IPTW (Lee and Little 2017; Robins et al. 2000). We conducted a multilevel analysis without propensity score weighting and used the likelihood ratio test to determine the best-fitting multilevel model. We used the *margins* command in Stata software version 15 to first estimate the predicted probabilities (Muller and MacLehose 2014) of an illness, severe illness episode, and the use of healthcare services, and then estimated the average marginal effects (AME) of residing in the intervention district on these probabilities. Our threshold for statistical significance was set at a p value < 0.05. Given that joint hypotheses testing was not of interest in our study, we did not consider the use of Bonferroni correction in our analysis.

#### Estimating Effect on Inequalities in the Use of Health Services

To assess the effect of user fee removal on the inequalities in the use of health services, we used dummy variables for the district of residence and interaction terms for distance, SES, and illness severity. We conducted the nonlinear Wald-type test of homogeneity to assess interactions (Gould 1996). In order to interpret the associations more thoroughly, we used the *marginsplot* command in Stata (Williams 2015) to plot the relationship between the probability of using health services and user fee removal for distance, SES, and illness severity.

## Results

### Descriptive Statistics

Our study sample included 1123 comparison and 1040 intervention households. These 2163 households included 2779 children under 5 years of age (1476 children in comparison and 1303 children in intervention districts). Approximately one quarter of children (24.6%) in the intervention district experienced an illness episode compared to one-fifth (19.9%) in the comparison district. Among the children who experienced an illness episode, 66.8% in the intervention were severe, compared with 71.3% in the comparison district. 45.1% of those who experienced an illness episode used the health service in the intervention district, compared to 18.0% in the comparison district.

### Propensity Score Weighting

As displayed in Table 1, before weighting, the standardized mean differences were greater than 0.15 for 7 variables. After weighting, the values of the standardized effect size (std.eff.sz) were attenuated and fell to almost null for all covariates with values lower than 0.02.

**Table 1:** Characteristics between intervention and comparison group on all covariates before and after propensity score weighting

	Intervention	Comparison	std.eff.sz	Intervention	Comparison	std.eff.sz
Age						
0–1 year	24.1	20.6	0.08	22.4	22.3	0.00
l–2 years	20.0	18.9	0.03	19.3	19.1	0.01
2–3 years	16.2	19.7	− 0.1	18.1	18.2	0.00
3–4 years	23.8	18.8	0.12	21.3	20.9	0.01
4–5 years	15.9	22.1	− 0.17	18.9	19.5	− 0.01
Sex						
Male	52.3	49.9	0.05	51.4	50.8	0.01
Female	47.7	50.0	− 0.05	48.6	49.2	− 0.01
Residence						
Rural	89.5	90.8	− 0.04	89.9	89.8	0.01
Urban	10.5	9.3	0.04	10.0	10.2	− 0.01
Distance						
0–5 km	46.9	42.5	0.09	44.3	44.9	− 0.01
5–10 km	32.9	30.4	0.05	31.9	30.9	0.02
10–15 km	20.2	27.1	− 0.17	23.7	24.1	− 0.01
Socio-economic status						
Poor	26.0	19.2	0.16	22.1	21.4	0.02
Middle	54.0	63.3	− 0.19	59.3	60.3	− 0.02
Wealthiest	19.9	17.5	0.06	18.6	18.3	0.01
1 child	17.3	24.8	− 0.2	21.7	21.8	0.00
2 children	37.8	47.7	− 0.2	42.7	43.0	− 0.01
≥ 3 children	44.9	27.6	0.35	35.6	35.2	0.01
Mother has attended school						
No	90.3	92.1	− 0.06	91.7	91.5	0.01
Yes	9.7	7.9	0.06	8.3	8.6	− 0.01
Slept under bednet last night						
No	13.4	13.4	0.00	13.5	13.4	0.00
Yes	86.7	86.7	− 0.00	86.6	86.6	0.00
Access to potable water						
No	60.7	76.9	− 0.33	68.8	70.1	− 0.03
Yes	39.3	23.1	0.34	31.2	29.9	0.03
Mean distance to health center						
0–5 km	54.3	48.6	0.11	51.2	51.4	0
5–10 km	30.1	30.5	− 0.01	30.2	30.3	0
> 10 km	15.7	20.9	− 0.14	18.6	18.4	0.01
Village level SES						
Poor	7.8	9.4	− 0.06	7.5	8.3	− 0.03
Middle	80.7	82.4	− 0.04	82.8	82.7	0
Wealthiest	11.5	8.2	0.11	9.7	9.1	0.02

### Main Effect Estimates

The likelihood ratio (Table 2) demonstrated that GBM propensity weighting combined with multilevel modelling provided a better fit than the simple multilevel model (LR χ^2^(2) = − 2.15; P > χ^2^ = 1.00). The probability of an illness recall was 4.44% (95% CI 1.0–9.8) higher among children living in the intervention district compared to children living in comparison district, although this marginal effect was not statistically significant. However, in the case of illness, children living in intervention district increase their probability of using a health facility by 17.2% (95% CI 15.0–26.6), compared with children living in comparison district.

**Table 2:** Predicted probabilities of an illness episode and use of health services according to distance, SES and illness severity (95% confidence interval) [Comparing Simple Multilevel regression with boosted propensity score with IPTW]

	Simple multilevel		Boosted PS with IPTW		Difference between the predicted probabilities Intervention versus Comparison		Test of homogeneity^a^
	Intervention	Comparison	Intervention	Comparison	Simple multilevel	Boosted PS with IPTW	
Illness episode	24.3 (20.3–28.2)	19.9 (16.2–23.7)	24.3 (20.5–28.2)	19.8 (15.9–23.7)	4.3 (− 1.1–9.8)	4.4 (− 1.0–9.8)	
Distance							
0–5 km	27.4 (21.8–32.9)	20.4 (14.8–26)	28.0 (22.01–34.1)	20.3 (13.5- 27.02)	7.7 (0.0–15.5)	7.9 (− 1.2–16.9)	
5–10 km	22.1 (16.4–27.7)	18.8 (13.3–24.3)	22.9 (16.9–28.9)	18.8 (12.1–25.6)	3.9 (− 4.1–11.9)	4.1 (− 4.8–13.1)	p = 0.56
10–15 km	19.3 (12.7–25.9)	21.07 (14.7–27.3)	19.2 (12.2–26.2)	20.2 (16.3–24.1)	− 1.2 (10.4–8)	− 1.0 (− 9.0–7.1)	
Socio-economic status							
Poor	20.9 (15.5–26.4)	20.8 (15.0–26.5)	20.6 (13.9–27.3)	21.1 (14.5–27.6)	0.5 (− 7.4–− 8.4)	− 0.6 (− 9.9–8.7)	
Middle	25.5 (20.9–30.1)	18.8 (14.9–22.8)	26.3 (22.2–30.3)	18.2 (14.3–22.1)	7 (0.9–13)	7.9 (2.2–13.6)	p = 0.05
Wealthiest	23.99 (18.1–29.9)	23.6 (17.3–30.0)	23.8 (17.9–29.7)	23.8 (17.2–30.4)	0.7 (− 8.0–9.4)	− 0.2 (− 9.0–8.6)	
Use of health service	44.2 (37.8–50.5)	24.90 (18.8–30.9)	42.3 (35.2–49.4)	24.8 (17.9–31.6)	19.2 (10.4–28.1)	17.2 (15.0–26.6)	
Distance							
0-< 5 km	50.9 (42.5–59.3)	33.5 (23.1–44.0)	50.9 (39.5–62.4)	33.9 (21.9–45.82)	17.5 (4–30.9)	16.8 (0.9–32.7)	
5–10 km	34.3 (23.9–44.6)	16.2 (7.32–25.2)	31.6 (20.8–42.4)	15.9 (8.1–23.8)	18 (4.2–31.9)	15.4 (2.3–28.4)	p = 0.02
10–15 km	41.1 (27.1–55.1)	16.1 (7.5–24.6)	36.9 (25–48.8)	15.9 (5.1–26.8)	25.1 (8.4–41.8)	20.6 (5.2–36.1)	
Socio-economic status							
Poor	37.0 (24.9–49.1)	22.4 (11.3–33.4)	34.0 (14.8–53.2)	22.4 (10.1–34.7)	13.8 (− 2.9–30.6)	11.1 (− 10.8–33.0)	
Middle	46.6 (38.9–54.2)	21.9 (14.9–29.1)	44.8 (37.2–52.4)	23.3 (14.1–32.5)	23.8 (13.1–34.5)	21.3 (9.9–32.8)	p = 0.61
Wealthiest	45.2 (33.9–56.5)	31.2 (19.3–43.0)	43.3 (31.2–55.5)	30.4 (16.1–44.6)	13.1 (− 3.5–29.7)	12.4 (− 5.9–30.7)	
Illness severity							
No	25.6 (17.3–34.0)	13.9 (6.7–21.0)	23.4 (15.5–31.4)	13.4 (5.7–21.1)	10.3 (− 0.9–21.5)	8.8 (− 0.2–19.6)	
Yes	53.8 (46.4–61.2)	28.8 (21.7–35.9)	51.2 (43.0–59.5)	30.7 (22.8–38.5)	22.9 (12.2–33.7)	20.7 (9.9–31.5)	p = 0.00

	AIC		BIC		
Simple multilevel	696.1		784.4		
Boosted PS with IPTW	689.9		769.4		LR χ^2^(2) = − 2.15 p > χ^2^ = 1.0000

^a^Test for interaction assessing whether the average marginal effects distance, SES, illness severity differed across levels of these variables

### Assessing Effect Modification by Distance, Illness Severity and Household SES

**Fig. 1:**
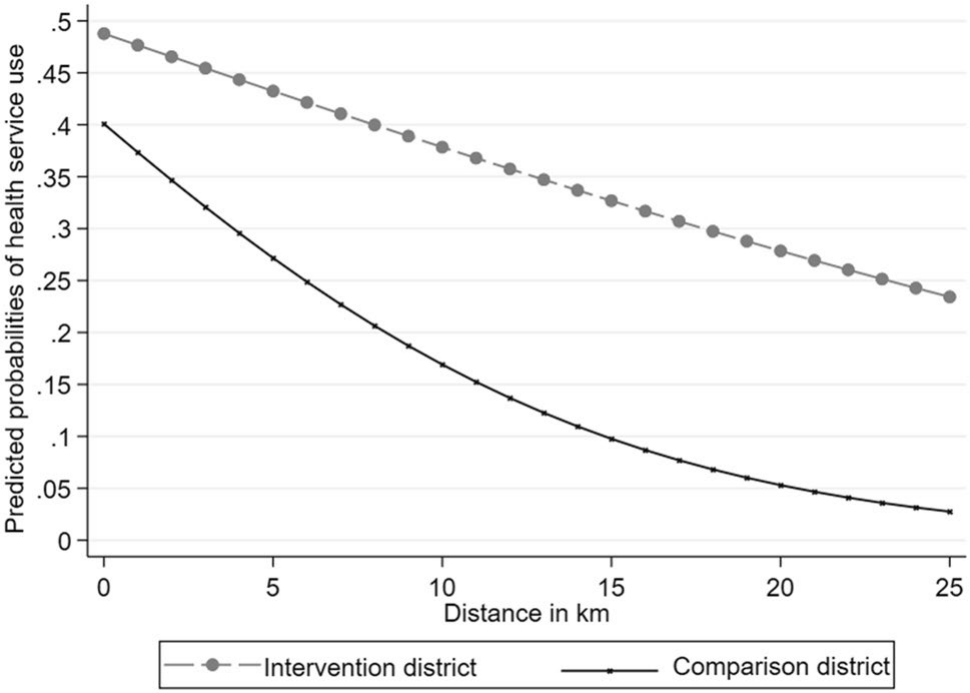
Predicted probabilities of health service use by distance to health facility, by district

As shown in Fig. 1 and Table 2, the use of services was higher at any distance for children in the intervention district compared to those living in the comparison district.

However, the homogeneity test indicated that children who live within 5 km continued to use health centers more often than those living more than 5 km away in both intervention and comparison groups  (Table 2) [χ^2^(5) = 12.90, p = 0.02]. Moreover, the effect of distance on the probability of seeking care was different between the intervention and comparison group. As the distance between households and health centers increases, the probability of using health services decreases dramatically faster in logarithmic terms in the comparison district, whereas the decrease was linear and less steep in the intervention district. In addition, for an episode of severe illness, although children in both groups increased their probability of use of health services when compared to those without severe illness [χ^2^(3) = 37.49, p = 0.00], the probability of use of health services for severe illness was 20.3% (95% CI 9.9–31.5) higher for children living in the intervention district.

**Fig. 2:**
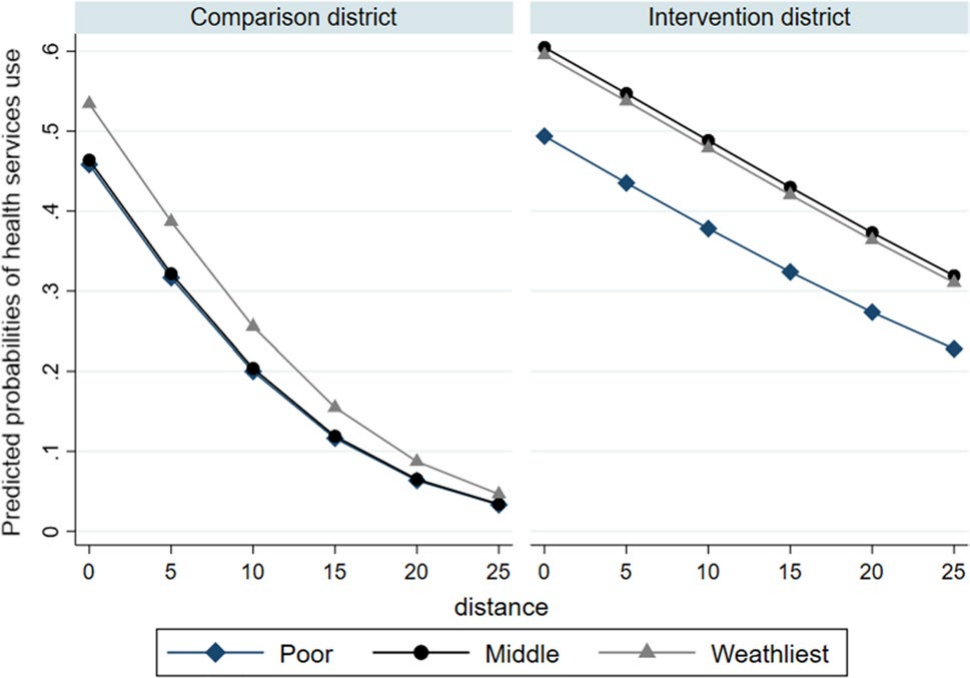
Predicted probabilities of health services use in the event of severe illness stratified by distance and household wealth

Furthermore, when we compared children living in the intervention district to those living in the comparison district, the use of health services increased across all socioeconomic groups ranging from 11.1% (95% CI − 10.8–33.0) to 21.3% (95% CI 9.9–32.8). However, no statistically significant differences were found between socioeconomic groups [χ^2^(5) = 12.90, p = 0.61]. Finally, Fig. 2 demonstrates that the probability of using health services in the event of a severe illness did not significantly differ across SES groups.

## Discussion

Our findings provide insight into how user fee removal may be associated with the probability of an illness, although in our study, no significant differences were detected with self-reported morbidity in children under 5. Indeed, we found that for children under 5, living in an intervention district was associated with an increased probability of health service use, particularly when the illness episode was severe. This association held true irrespective of the distance to the health facility and household SES, which is also consistent with prior evidence (Lagarde and Palmer 2011; McKinnon et al. 2014; Ridde et al. 2013a, b; Zombré et al. 2017b). In comparison to prior studies, the added value of our analysis lies in its capacity to quantify the magnitude in terms of probabilities, suggesting that years after user fee removal onset, the intervention was associated with a 17.2% increase in the probability of seeking care in the case of illness.

Despite the increase in the use of health services, we found that residing in a district where user fees have been removed was not associated with any reduction in the probability of an illness occurring. This finding is consistent with a randomized controlled trial based in Ghana, which analyzed the effect of user fee removal on self-reported and clinical child health outcome, one year after implementation (Ansah et al. 2009). One possible explanation of this result may be related to the fact that we used intention to treat estimation to derive the probability of an illness occurring, without considering the mediating effect of the intensity of health service use nor the quality of care received. Another explanation for the null findings could be that in the context of Burkina Faso, the healthcare system would explain a small part of the morbidity variance alongside other important social determinants of morbidity that were not included in our study such as lifestyle, nutrient deficiency, and sanitation (Mosley and Chen 1984; Victora et al. 1997). Alternatively, the medical care provided in the intervention district could have had no measurable effect on morbidity, as measured by our study.

In line with prior evidence (Heinmüller et al. 2012; Ridde et al. 2013a, b), the probability of health service use was 20.3% higher with severe illness. This finding is consistent with overall health seeking literature; the more severely ill the person is, the more likely they are to seek care (De Allegri et al. 2011; Rutebemberwa et al. 2009; Rutherford et al. 2010). This highlights the potential of user fee removal in reducing inequalities in access according to the severity of illness. Additionally, we found no statistically significant differences in the probability of using health services according to the socio-economic status nor in the event of a severe illness episode in children across different SES groups.

Finally, our findings showed that the impact of distance on the use of services is less dramatic for children living in the intervention district compared to those living in the comparison district, meaning that families living further away in the intervention district were less likely to ration their use of health services than those who live further away. This supports the findings of a previous study (Ridde et al. 2013a, b) in that the user fee removal dilutes the impact of distance on the use of services.

### Strengths and Limitations

Our findings contribute to the ongoing debate on whether user fee removal improves the use of health service in the longer term and whether it contributes to a reduction in morbidity. We relied on propensity scores methods (McCaffrey et al. 2004) to counteract the lack of baseline data and to reduce sample selection bias. Often NGO pilot projects do not implement baseline surveys due to cost restraints (Ridde 2015) and our study is based upon rigorous analyses despite this baseline limitation. We also used predicted probabilities and graphical analyses to thoroughly examine the impact of interaction on the magnitude of the intervention.

Despite our rigorous analysis, our study has a number of limitations. As our study only measured outcomes at one point in time years after the intervention onset and did not include baseline data, our study did not capture changes over time. Given this, the results must be interpreted with caution. We also acknowledge limitations in using propensity score weighting such as the possibility of children from the intervention and comparison districts differing in unknown village and individual-level confounders or unknown effect modifiers. Furthermore, factors than user fee removal could have differentially affected the children’s probability of health service use and probability of illness over time.

The effect estimates may have been influenced by a reporting bias or misclassification of the illness episode and the use of health services, as parents’ self-reporting of their children’s illness is often related to socio-economic status, levels of education, availability of health facilities, and public information on illness and treatment (Sen 2002). This could have led to an underestimation of the protective effect of being exposed to user fee removal on the probability of an illness. Moreover, our analyses were unable to account for a possible reverse causation due to the fact that health service use may reduce the risk of morbidity, in turn possibly reducing the likelihood of using healthcare services. If present, this possible reverse causation may have diluted intervention effects, as we used prevalent cases of illnesses in our cross-sectional study. Future studies should consider this potential effect by using structural equation modeling or an instrumental variable approach based on a natural experiment (Glymour 2017) with the user fee removal as an instrument to analyse the effect of health service use on child health outcomes. Finally, as the impact of user fee removal will vary among communities, depending on the particular structural, cultural, and environmental contexts, the results of our study should not be generalized.

## Conclusion

Our findings add to the body of research on user fee removal effectiveness in healthcare use and morbidity in the longer term. Based upon our results, there was a significant increase in the use of health services although this did not translate into a reduction on the risk of illness occurrence nor on the differences in health service access based upon socioeconomic status. As Burkina Faso launched a nation-wide user fee removal policy for children in 2016, our findings contribute to highlighting the limitations of focusing efforts on user fee removal policies in order to improve child health outcomes and the need for synergistic health policies to improve child health outcomes within the prospect of the universal coverage.
